# Distress among healthcare providers who provided end-of-life care during the COVID-19 pandemic: a longitudinal survey study (the CO-LIVE study)

**DOI:** 10.1186/s12904-024-01446-y

**Published:** 2024-05-28

**Authors:** Masha S. Zee, Bregje D. Onwuteaka-Philipsen, Erica Witkamp, Benthe Heessels, Anne Goossensen, Ida J. Korfage, Yvonne N. Becqué, Corine Nierop-van Baalen, Agnes van der Heide, H. Roeline Pasman

**Affiliations:** 1 Department of Public and Occupational Health, Expertise Center for Palliative Care, Amsterdam UMC, VU University, Van der Boechorststraat 7, 1081 BT Amsterdam, The Netherlands; 2 Department of Public Health, Erasmus MC University Medical Center, Rotterdam, The Netherlands; 3 Research Center Innovations in Care, Rotterdam University of Applied Sciences, Rotterdam, The Netherlands; 4 University of Humanistic Studies, Utrecht, The Netherlands

**Keywords:** COVID-19 pandemic, End-of-life care, Healthcare providers, Palliative care

## Abstract

**Introduction:**

The COVID-19 pandemic had a significant impact on care at the end-of-life due to restrictions and other circumstances such as high workload and uncertainty about the disease. The objective of this study is to describe the degree of various signs experienced by healthcare providers throughout the first 18 months of the pandemic and to assess what provider’s characteristics and care circumstances related to COVID-19 are associated with distress.

**Methods:**

A longitudinal survey study among healthcare providers from different healthcare settings who provided end-of-life care during the pandemic’s first 18 months. Data of four time periods were analyzed using descriptive statistics, logistic regression analysis and Generalized Estimating Equation.

**Results:**

Of the respondents (*n*=302) the majority had a nursing background (71.8%) and most worked in a hospital (30.3%). Although reported distress was highest in the first period, during the first wave of COVID-19 pandemic, healthcare providers reported signs of distress in all four time periods. Being more stressed than usual and being regularly exhausted were the most common signs of distress. Healthcare providers working in nursing homes and hospitals were more likely to experience signs of distress, compared to healthcare providers working in hospice facilities, during the whole period of 1.5 years. When HCPs were restricted in providing post-death care, they were more likely to feel more stressed than usual and find their work more often emotionally demanding.

**Conclusion:**

A substantial amount of healthcare providers reported signs of distress during the first 1.5 years of the COVID-19 pandemic. A cause of distress appears to be that healthcare providers cannot provide the care they desire due the pandemic. Even though the pandemic is over, this remains an important and relevant finding, as high workload can sometimes force healthcare providers to make choices about how they provide care. Given that this can cause prolonged stress and this can lead to burnout (and HCPs leaving their current positions), it is now especially important to continue observing the long term developments of the well-being of our healthcare providers in palliative care and provide timely and adequate support where needed.

**Supplementary Information:**

The online version contains supplementary material available at 10.1186/s12904-024-01446-y.

## Introduction

The COVID-19 pandemic had a significant impact on healthcare from 2020 until 2023 on the way healthcare services were provided. There was a lot of uncertainty about the course of the disease, the pandemic and the strategies to manage it. Additionally, healthcare and its workers faced high pressure, alongside occasional implementation of severe restrictions, such as wearing personal protective equipment (PPE) and limiting contact with patients and relatives, to prevent the spread of the COVID-19 virus [1–3]. Research indicates that healthcare providers experienced increased levels of stress, anxiety, and fatigue during the COVID-19 pandemic [4], with many of them reporting symptoms of burnout, anxiety, or depression [5].

Healthcare providers who were specifically involved in end-of-life encountered circumstances that disrupted the normal process of end-of-life care [1, 2, 6–8]. The pandemic not only altered how care was delivered to patients nearing the end of their life but also presented unique challenges for the well-being of healthcare professionals.

Limitations in how care was delivered to patients approaching the end of life led to moral distress because it conflicted with their professional standards. Due to the visiting restrictions, social distancing and staff shortages, health care providers (HCPs) expressed concerns about their ability to provide compassionate and empathetic care to patients and their families as they did before the pandemic [9]. This concern is supported by a review on end-of-life care in nursing homes during the COVID-19 pandemic that described that the most common emotions experienced by HCPs were fear, depression, stress, anxiety, hopelessness, and grief. This was partly attributed to the perception among healthcare providers that they were not able to maintain the same standard of care, as compared to before the pandemic given the circumstances of the pandemic [10]. Furthermore, in a study on moral distress among 120 palliative care and hospice social workers from the United States, several causes of such moral distress were identified, including limitations in visiting options for relatives and the need to wear protective equipment which hindered personal contact and communication [11]. A study on the mental health of 142 palliative care professionals during the COVID-19 pandemic Hong Kong described that palliative care professionals experienced stress (82%), at least mild depression (42%), anxiety symptoms (43%), and post-traumatic stress symptoms (60%) [12]. Moreover, a Italian study among home 145 palliative care clinicians investigated the difference in symptoms of burnout between the first wave of the pandemic and one year later. After a year, the number of healthcare providers experiencing burnout remained roughly the same, but there was a higher level of emotional exhaustion [13].

Studies on the well-being of HCPs that provided end-of-life care during the pandemic mostly had cross-sectional designs and focuses on the initial months of the pandemic. However, there is a gap in understanding the ongoing distress experienced by HCPs over the course of the pandemic beyond these initial months. Additionally, limited information is available about how factors related to COVID-19, such as visiting restrictions, influenced distress of HCPs over time in different settings. The objective of this study is to describe the degree of various signs of distress (stress, exhaustion, considering work emotionally and physically demanding and the need for emotional support) experienced by HCPs throughout the first 18 months of the pandemic. Furthermore, this study also aims to assess what provider’s characteristics (setting and profession) and care circumstances related to COVID-19 (restrictions regarding visits and post-death care and a scarcity of PPE) are associated with distress. Insight in this helps to better understand the long lasting impact the COVID-19 pandemic had on the well-being of HCPs that provided end-of-life care in different settings.

## Methods

### Design

An observational longitudinal online survey study was conducted as part of the CO-LIVE study. CO-LIVE is a mixed methods study into the experiences of bereaved relatives and HCPs that provided end-of-life care during the COVID-19 pandemic [6].

### Population & data collection

Data was collected from a convenience sample of HCPs that provided end-of-life care (for both COVID and non-COVID patients) during the initial 18 months of the COVID pandemic (March 2020 – September 2021). These HCPs came from various professions and from different settings in the Netherlands.

**Fig. 1:**
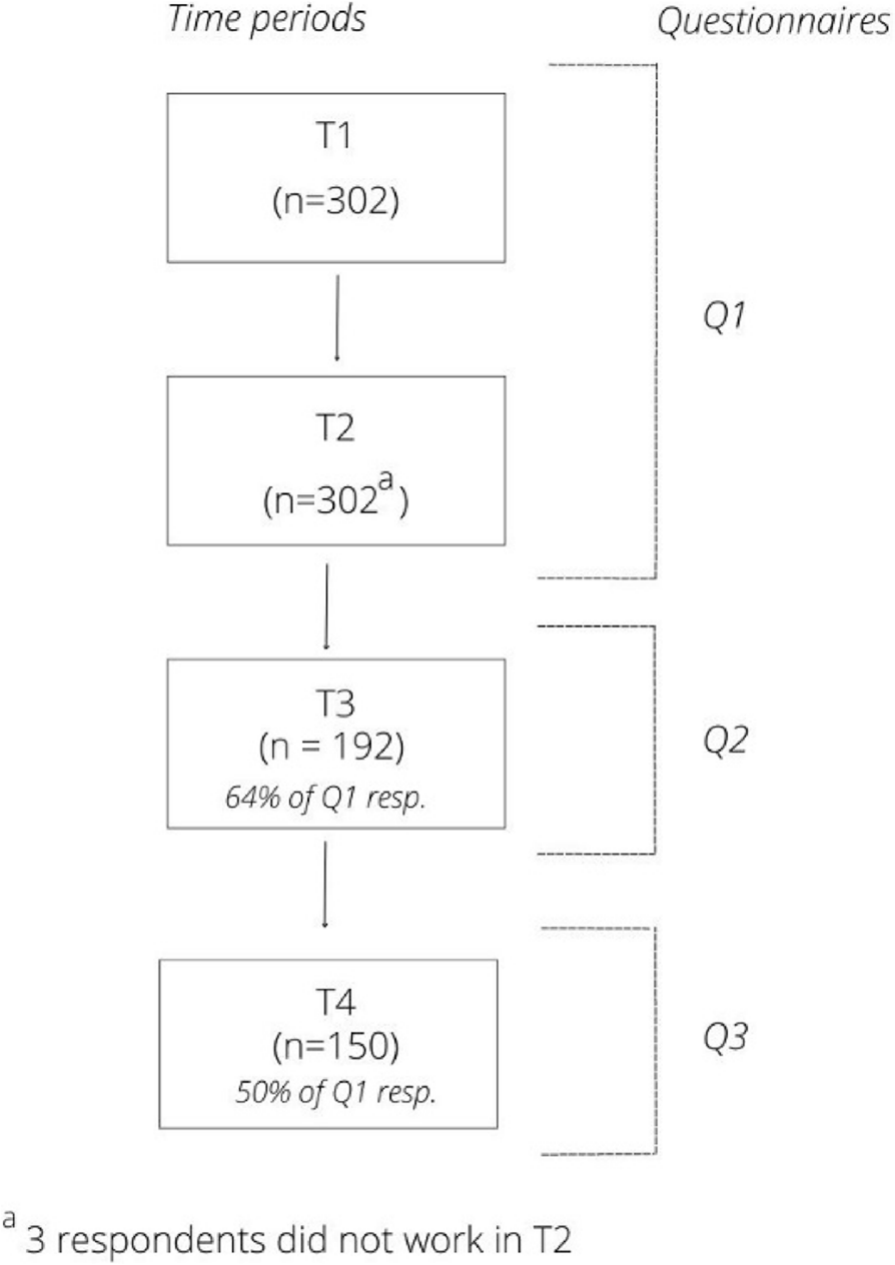
Respondents per time period and questionnaire

**Fig. 2:**
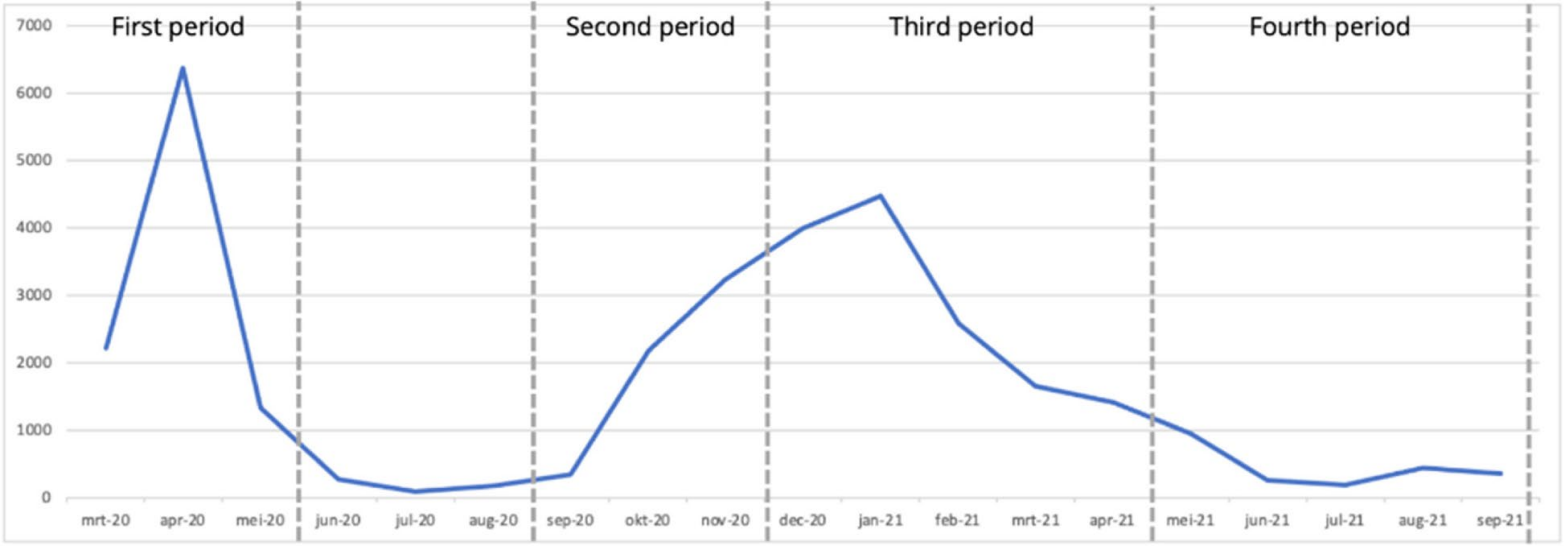
COVID-19 deaths in the Netherlands, march 2020 – September 2021

Data collection covered four time periods, with three questionnaires, Q1, Q2 and Q3 (Fig. 1). Q1 was distributed in November 2020 and contained questions about two time periods: March 2020 – May 2020 (T1) and September 2020 to November 2020 (T2). These periods are considered to be the first wave (T1) and the start of the second wave (T2) of the COVID-19 pandemic in the Netherlands [14, 15]. Q2 was distributed in April 2021 and concerned the period between December 2020 – April 2021 (T3). Q3 was distributed in September 2021 and concerned the time period between May 2021 and September 2021 (T4). Figure 2 shows the number of people that died of COVID-19 in the Netherlands within the four time periods. This provides context about the severity of the pandemic in these researched time periods [16].

Invitations for questionnaire 1 (Q1) were sent to end-of-life care providers that had participated in a previous part of the CO-LIVE study [6]. Additional respondents for Q1 were recruited via (social) media. When respondents indicated in the Q1 questionnaire that we could approach them for another questionnaire, they received an invitation to Q2 and Q3. No other respondents were recruited for Q2 and Q3. Furthermore, respondents of Q1 who missed Q2 could participate in Q3. All questionnaires were designed and distributed via questionnaire software Survalyzer.

### Measurements &analysis

Respondents answered questions regarding the context in which they provided care and about distress in each time period (T1 – T4) during the COVID-19 pandemic. Questions related to COVID-19 and questions related to distress were self-developed and were based on the situation surrounding the pandemic, existing literature and insights gathered from interviews with HCPs about end-of-life care during the pandemic (Appendix 1).

We included five statements about distress and asked HCPs to what extent they agreed with the statements on a 5-point scale. HCPs were asked whether they were more stressed than usual, if they considered their work emotionally and physically demanding and if they felt exhausted regularly. Answer options were dichotomized to ‘agreed’ (i.e. ‘agree’ and ‘strongly agree) and ‘not agreed’ (i.e. ‘neutral’ ‘disagree’ and ‘strongly disagree’). We also included a question about how much emotional support they needed during these time periods; answer options were dichotomized to ‘more than usual’ (i.e. ‘more than usual) and ‘not more than usual’ (i.e. ‘as much as usual’ and ‘less than usual’).

Characteristics of respondents included gender, age, profession and setting. Setting was categorized into: home, nursing home, hospice facility, other (including for example a GP practice or institutions for people with intellectual disabilities) and multiple settings. Profession of HCPs was divided in three categories; nurses (including registered nurses, nursing aids and nurse practitioners), physicians (e.g. general practitioners, pulmonary and geriatric physicians) and other (e.g. spiritual counselors, paramedics and volunteers).

The circumstances of care related to COVID-19 consisted of three different variables; visit restrictions, the availability of PPE and restrictions in post-death care. Visit restrictions in the last days of the patients’ lives were dichotomized to ‘yes’ for any type of visiting restrictions (maximum persons allowed, maximum time for visits) and ‘no’ for none. HCPs were asked if there were restrictions in post-death care (e.g. not being allowed to take care of the deceased patients’ body) (yes/no). Post-death care is primarily administered by the same healthcare professionals who cared for the patient prior to their passing. We asked HCPs if there was enough PPE available and dichotomized the answers to yes (‘yes’) and no (‘no’ ‘no, not always’, ‘no not enough for everyone who needed it’). This item was not included in Q3, since there was no longer a national shortage of PPE in T4 [17]. We included the item on T4 in the analysis and indicated that there were no shortages.

IBM SPSS statistics 28 and Stata 17 were used to analyze the data. Characteristics from HCPs and COVID-19 related circumstances of care were described to summarize the data per time period. General Estimating Equations (GEE) were used to study differences between time periods and to investigate what COVID-19 related circumstances of care and characteristics of respondents (as independent variables) are associated with the statements about distress (dependent variables). The GEE accounted for clustering of within-subject data (up to four measurements over time per individual). A univariate analysis was done with all independent variables. When independent variables were associated with the dependent variable (p<0.10), the variables were entered in the multivariable regression analysis. This also applied to the logistic regression analysis that was used to investigate the associations between circumstances of care and HCP characteristics per time period.

### Ethics

For every questionnaire, study information was provided and prior to filling in the questionnaire and the respondents were asked for consent to participate. The Medical Research Ethics Committee of the Erasmus MC in Rotterdam, the Netherlands determined exception from formal review under Dutch law (MEC-2020-0254).

## Results

### Characteristics of healthcare providers

Data of 302 (T1), 299 (T2), 192 (T3) and 150 (T4) respondents is included (Table 1). The characteristics of the respondents in the different time periods are described in Table 1. Most respondents were women (87.2 – 90.1% and between 46-60 years of age (44.9 - 55.8%). Over half of the respondents had a nursing background (61.6 – 71.8%) and about one in third worked in a hospital (27.4 – 30.3%).)

**Table 1:** Characteristics of HCPs and end-of-life care during four different time periods (absolute numbers and percentage)

	**T1**	**T2**	**T3**	**T4**
	**Q1**^**a**^		**Q2**	**Q3**
	*N* = 302		*N* = 192	*N* = 150
	N (%)		N (%)	N (%)
**Gender**				
Men	32 (10.8)		19 (9.9)	19 (12.8)
Women	265(89.2)		173 (90.1)	129 (87.2)
**Age**				
<35 years	61 (20.7)		30 (15.9)	13 (8.8)
36-45 years	58 (19.7)		29 (15.3)	23 (15.6)
46-60 years	132 (44.9)		96 (50.8)	82 (55.8)
>60 years	43 (14.6)		34 (18.0)	29 (19.7)
**Profession**				
Nurse	216 (71.8)		129 (68.8)	90 (61.6)
Physician	40 (13.2)		24 (12.8)	22 (15.1)
Other	45 (15.0)		35 (18.6)	34 (23.4)
**Setting**				
Home	47 (15.8)		33 (17.6)	20 (13.7)
Nursing home	64 (21.5)		34 (18.1)	29 (19.9)
Hospital	90 (30.3)		53 (28.2)	40 (27.4)
Hospice facility	54 (18.2)		38 (20.2)	29 (19.9)
Other	17 (5.7)		13 (6.9)	15 (10.3)
Multiple	25 (8.4)		17 (9.0)	13 (8.9)

Number of missing observations range (over Q1-Q3): gender (0-5), age (3-8), profession (0-4), setting (3-5)

^a^Q1 was distributed in T2 and contained questions about both T1 and T2

### Signs of distress during the COVID-19 pandemic over time

**Fig. 3:**
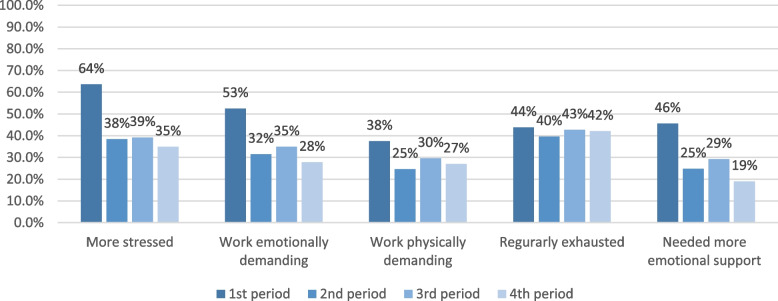
Signs of distress over four time periods (% who agreed with the statement)

The signs of distress and their development and differences over time are shown in Fig. 3 and Table 2. For all statements except the statement about exhaustion, the percentages of HCPs that agree significantly decreased in T2, slightly increased in T3 and decreased again in T4. This was different for the statement about exhaustion where the percentages did not decline significantly and ranged between 39.6% and 43.8% during all four time periods throughout the 18 months.

**Table 2:** Signs of distress among HCPs in four time periods (percentages and Odds Ratio’s (OR’s) with 95% confidence intervals (CI’s))

	**First period**		**Second period**		**Third period**		**Fourth period**	
	% (CI)	OR (CI)	% (CI)	OR (CI)	% (CI)	OR (CI)	% (CI)	OR (CI)
More stressed	63.6% (57.8-69.2)	ref	38.4% (32.8-44.2)	**0.35 (0.27- 0.47)**	39.2% (32.1-46.6)	**0.37 (0.25- 0.54)**	34.9% (27.0-43.5)	**0.31 (0.21-0.47)**
Work emotionally demanding	52.5% (46.6-58.4)	ref	31.5% (26.3-37.2)	**0.41 (0.31- 0.55)**	34.9% (28.0-42.3)	**0.49 (0.34- 0.70)**	27.8% (20.5-36.0)	**0.37 (0.25-0.54)**
Work physically demanding	37.5% (31.9-43.3)	ref	24.6% (19.8-30.0)	**0.56 (0.43-0.72)**	29.6% (23.1-36.8)	0.77 (0.53- 1.11)	27.0% (19.8-35.2)	0.65 (0.42-1.00)
Regularly exhausted	43.8% (38.0-49.7)	ref	39.6% (33.9-45.3)	0.85 (0.68-1.08)	42.7% (35.5-50.2)	1.00 (0.69- 1.44)	42.1% (33.7-50.8)	0.96 (0.65-1.43)
Needed more emotional support	45.6% (39.8-51.5)	ref	24.8% (20.0-30.2)	**0.39 (0.29- 0.53)**	29.2% (22.8-36.4)	**0.48 (0.33- 0.71)**	19.0% (12.9-26.6)	**0.28 (0.17-0.46)**

Number of missing observations range from: T1 (0-2), T2 (0-2), T3 (0-2), T4 (0-2)

Bold ORs indicate that they are significantly different compared to T1

HCPs were significantly more likely to be more stressed than usual, find their work emotionally demanding and need more support than usual in T1 compared to T2 (ORs 0.35 – 0.41), T3 (ORs 0.37 – 0.49) and T4 (ORs 0.28 – 0.37). Furthermore, HCPs were significantly more likely to find their work physically demanding in T1 compared to T2 (OR 0.56). The second, third and fourth period did not show any significant differences in odds of feeling regularly exhausted compared to the first period.

### Care circumstances related to COVID-19

Table 3 shows how the circumstances of care related to COVID-19 changed over the four time periods. Visit restrictions became less present over time (from 91.1% in T1 to 56.3% in T4). The percentage of HCPs that did not have enough PPE was especially high in the first period (T1: 52.3%), but declined quickly in the next time periods. Over time, more HCPs were more often allowed to provide post-death care (T1: 80.5%; T4: 96.6%). For all COVID-related circumstances of care the difference between T1 and T4 is significant (Chi-square test: *p*<0.001).

**Table 3:** Care circumstances related to COVID-19 during four time periods

	**T1**	**T2**	**T3**	**T4**
	*N*=302	*N*=299	*N*=192	*N*=150
	N(%)	N (%)	N (%)	N(%)
**Visit restrictions**				
Visit restrictions in place	275 (91.1)	246 (82.3)	152 (80.9)	80 (56.3)
No visit restriction in place	27 (8.9)	53 (17.7)	36 (19.1)	62 (43.7)
**PPE**				
Enough PPE available	144 (47.7)	279 (93.3)	177 (94.1)	150 (100)
Not enough PPE available	158 (52.3)	20 (6.7)	11 (5.9)	0 (0.0)
**Post-death care**				
Allowed to provide post-death care	243 (80.5)	261 (87.3)	170 (90.4)	141 (96.6)
Not allowed to provide post-death care	59 (19.5)	38 (12.7)	18 (9.6)	5 (3.4)

number of missing observations range (over T1-T4): visit restrictions (0-8), enough PPE (0-4), allowed to provide post-death care (0-4)

### Associations between signs of distress and HCP characteristics and circumstances of care related to COVID-19 during all four time periods

Table 4 shows the results of the GEE analysis to identify associations between signs of the distress and the characteristics of HCPs and the circumstances of care during all four time periods. Compared to nurses, physicians (OR 0.22 ) and other HCPs (OR 0.42 ) were less likely to find their work physically demanding.

**Table 4:** Associations between characteristics of HCPs and circumstances of care, and signs of distress during all four time periods (ORs and 95% confidence intervals)^*^

	**More stressed than usual**		**Emotionally demanding**		**Physically demanding**		**Regularly exhausted**		**Need more emotional support**	
	Univariate OR (CI)	Multivariate OR (CI)	Univariate OR (CI)	Multivariate OR (CI)	Univariate OR (CI)	Multivariate OR (CI)	Univariate OR (CI)	Multivariable OR (CI)	Univariate OR (CI)	Multivariable OR (CI)
**Gender**										
Women	1.00		1.00		1.00		1.00		1.00	
Men	0.87 (0.48 - 1.59)		0.64 (0.33 - 1.25)		1.33 (0.67 - 2.62)		0.69 (0.35 - 1.36)		0.69 (0.34 - 1.38)	
**Age**										
⩽35 years	1.00		1.00		1.00	1.00	1.00	1.00	1.00	
36-45 years	1.49 (0.86 - 2.59)		1.02 (0.57 - 1.83)		0.70 (0.39 - 1.26)	0.83 (0.45 - 1.52)	1.35 (0.77 - 2.36)	1.32 (0.75 - 2.34)	1.22 (0.69 - 2.18)	
46-60 years	0.99 (0.62 - 1.59)		0.80 (0.50 - 1.30)		**0.57 (0.34 - 0.95)**	0.81 (0.47 - 1.38)	0.73 (0.45 - 1.17)	0.87 (0.53 - 1.44)	0.82 (0.51 - 1.31)	
>60 years	0.84 (0.46 - 1.55)		1.01 (0.56 - 1.82)		**0.56 (0.29 - 1.08)**	1.01 (0.50 - 2.03)	**0.39 (0.20 - 0.77)**	0.50 (0.24 - 1.04)	0.75 (0.39 - 1.45)	
**Profession**										
Nurse	1.00		1.00		1.00	1.00	1.00		1.00	1.00
Physician	1.35 (0.85 - 2.13)		0.62 (0.35 - 1.10)		**0.25 (0.13 - 0.50)**	**0.22 (0.11 - 0.46)**	0.71 (0.41 - 1.22)		**0.61 (0.34 - 1.10)**	0.57 (0.31 - 1.05)
Other	0.94 (0.65 - 1.36)		1.08 (0.74 - 1.59)		**0.40 (0.25 - 0.62)**	**0.42 (0.27 - 0.68)**	1.22 (0.82 - 1.80)		1.12 (0.73 - 1.72)	1.13 (0.74 - 1.74)
**Setting**										
Hospice	1.00	1.00	1.00	1.00	1.00	1.00	1.00	1.00	1.00	1.00
Home	**1.99 (1.05 - 3.79)**	**2.14 (1.13 - 4.05)**	1.16 (0.58 - 2.33)	1.22 (0.61 - 2.40)	1.13 (0.54 - 2.37)	1.04 (0.49 - 2.20)	**2.40 (1.07 - 5.34)**	2.25 (0.99 - 5.10)	1.50 (0.78 - 2.87)	1.51 (0.79 - 2.88)
Nursing home	**2.56 (1.44 - 4.54)**	**2.38 (1.32 - 4.30)**	**1.88 (1.10 - 3.22)**	**1.79 (1.05 - 3.05)**	**2.13 (1.15 - 3.94)**	**2.39 (1.24 - 4.60)**	**4.57 (2.39 - 8.71)**	**3.92 (2.05 - 7.50)**	**2.43 (1.39 - 4.25)**	**2.53 (1.43 - 4.50)**
Hospital	**2.59 (1.50 - 4.48)**	**2.44 (1.40 - 4.26)**	**1.83 (1.06 - 3.15)**	**1.77 (1.03 - 3.05**)	**2.26 (1.26 - 4.05)**	**2.25 (1.19 - 4.25)**	**4.77 (2.59 - 8.80)**	**3.90 (2.11 - 7.22)**	**2.10 (1.22 - 3.61)**	**2.18 (1.27 - 3.76)**
Other	1.23 (0.63 - 2.41)	1.25 (0.63 - 2.49)	1.19 (0.61 - 2.32)	1.17 (0.59 - 2.30)	0.95 (0.44 - 2.06)	1.29 (0.58 - 2.87)	**3.04 (1.35 - 6.88)**	**2.88 (1.26 - 6.61)**	1.33 (0.66 - 2.69)	1.47 (0.71 - 3.04)
More than 1	**2.09 (1.06 - 4.12)**	**2.03 (1.01 - 4.06)**	1.15 (0.52 - 2.54)	1.12 (0.51 - 2.45)	0.82 (0.38 - 1.75)	0.91 (0.41 - 2.04)	**2.85 (1.32 - 6.13)**	**2.66 (1.21 - 5.84)**	**1.92 (0.95 - 3.87)**	2.01 (0.99 - 4.06)
**Visit restrictions**										
Visit restrictions in place	1.00	1.00	1.00	1.00	1.00		1.00		1.00	
No visit restriction in place	**1.43 (0.99 - 2.09)**	1.39 (0.93 - 2.06)	**1.48 (1.00 - 2.17)**	1.38 (0.92 - 2.05)	1.21 (0.81 - 1.81)		1.28 (0.89 - 1.85)		1.37 (0.92 - 2.05)	
**PPE**										
Enough PPE available	1.00		1.00		1.00		1.00	1.00	1.00	
Not enough PPE available	1.19 (0.80 - 1.77)		1.13 (0.78 - 1.64)		1.06 (0.73 - 1.53)		**1.41 (0.99 - 2.00)**	1.45 (0.98 - 2.13)	1.08 (0.74 - 1.58)	
**Post-death care**										
Allowed to provide post-death care	1.00	1.00	1.00	1.00	1.00		1.00		1.00	1.00
Not allowed to provide post-death care	**1.76 (1.15 - 2.68)**	**1.61 (1.04 - 2.51)**	**1.59 (1.07 - 2.34)**	**1.52 (1.01 - 2.28)**	1.16 (0.78 - 1.74)		1.01 (0.71 - 1.46)		**1.43 (0.96 - 2.14)**	1.32 (0.87 - 2.00)
**Time**										
T1	1.00	1.00	1.00	1.00	1.00	1.00	1.00	1.00	1.00	1.00
T2	**0.38 (0.29 - 0.51)**	**0.57 (0.36 - 0.91)**	**0.43 (0.33 - 0.56)**	**0.61 (0.40 - 0.93)**	**0.61 (0.48 - 0.77)**	**0.62 (0.47 - 0.81)**	0.83 (0.67 - 1.03)	0.98 (0.71 – 1.35)	**0.43 (0.33 - 0.57)**	0.54 (0.34 - 0.84)
T3	**0.35 (0.25 - 0.49)**	**0.39 (0.27 - 0.55)**	**0.50 (0.37 - 0.67)**	**0.55 (0.40 - 0.75)**	**0.75 (0.55 - 1.02)**	0.74 (0.52 - 1.06)	0.95 (0.71 - 1.29)	1.19 (0.81 – 1.75)	**0.51 (0.38 - 0.69)**	0.51 (0.37 - 0.71)
T4	**0.31 (0.20 - 0.47)**	**0.48 (0.26 - 0.88)**	**0.33 (0.23 - 0.50)**	**0.50 (0.30 - 0.85)**	**0.60 (0.40 - 0.88)**	**0.62 (0.40 - 0.96)**	0.81 (0.57 - 1.15)	1.05 (0.67 – 1.63)	**0.31 (0.20 - 0.46)**	0.37 (0.21 - 0.65)

*N= up to four observations from 276 respondents; based on Generalized Estimated Equation

Bold values in the univariate analysis indicated OR’s that significantly differ from 1.00 (*p*<0.10)

Bold values in the multivariate analysis indicated OR’s that significantly differ from 1.00 (*p*<0.05)

HCPs working in hospitals and nursing homes, were more likely to experience all signs of distress than HCPs in hospices (ORs 1.77 – 3.90). In home care and in settings categorized as ‘other’, HCPs were more likely to feel more stressed than usual (OR 2.14 ) and to feel regularly exhausted (OR 2.25 ). HCPs who worked in multiple settings were also more likely to feel more stressed than usual (OR 2.03).

HCPs who were not allowed to provide post-death care, were more likely to feel more stressed than usual (OR 1.61 ) and to find their work emotionally demanding (OR 1.52).

### Associations per time period

Logistic regression analyses of the signs of distress per time period for the most common two signs of distress show some differences in associations between the time period (Table 5). During T2 HCPs aged between 36-45 years were more likely to feel more stressed than usual when compared to HCPs aged ⩽35 years (OR 2.30). HCPs aged >60 years were more likely to feel regularly exhausted compared to HCPs aged ⩽35 years in T1 (OR 0.35). Additionally, HCPs that had a profession categorized as ‘other’ , were more likely to be exhausted compared to nurses in T3 (OR 2.20).

**Table 5:** Associations between characteristics of HCPs and care and signs of distress (ORs and 95% confidence intervals) per time period

**More stressed than usual**								
	**T1**		**T2**		**T3**		**T4**	
	Univariate	Multivariate	Univariate	Multivariate	Univariate	Multivariable	Univariate	Multivariate
**Gender**								
Women	1.00		1.00		1.00		1.00	
Men	0.60 (0.28 – 1.25)		0.89 (0.42 - 1.89)		0.97 (0.36 - 2.59)		2.02 (0.75 - 5.49)	
**Age**								
⩽35 years	1.00		1.00	1.00	1.00	1.00	1.00	
36-45 years	0.85 (0.39 – 1.84)		**2.28 (1.06 - 4.92)**	**2.30 (1.05 - 5.03)**	**2.45 (0.86 - 6.98)**	2.19 (0.74 - 6.54)	1.56 (0.37 - 6.62)	
46-60 years	0.77 (0.40 -1.47)		1.53 (0.79 - 2.95)	1.72 (0.85 - 3.49)	0.95 (0.40 - 2.22)	1.08 (0.43 - 2.68)	0.54 (0.15 - 1.92)	
>60 years	0.83 (0.36 - 1.92)		0.86 (0.36 - 2.10)	1.10 (0.42 - 2.86)	0.53 (0.18 - 1.57)	0.65 (0.19 - 2.21)	0.44 (0.10 - 1.92)	
**Profession**								
Nurse	1.00		1.00		1.00		1.00	
Physician	1.11 (0.54 – 2.82)		1.46 (0.74 - 2.87)		**2.60 (1.04 - 6.49)**		0.89 (0.33 - 2.41)	
Other	0.61 (0.32 – 1.17)		0.86 (0.48 - 1.54)		1.18 (0.60 - 2.32)		1.14 (0.49 - 2.64)	
**Setting**								
Hospice	1.00	1.00	1.00	1.00	1.00	1.00	1.00	
Home	**2.84 (1.24 – 6.51)**	**3.56 (1.46 - 8.69)**	1.68 (0.73 - 3.86)	1.93 (0.80 - 4.67)	1.93 (0.64 - 5.82)	1.72 (0.55 - 5.36)	1.97 (0.54 - 7.16)	
Nursing home	**2.31 (2.09 – 4.87)**	**1.98 (0.92 - 4.25)**	**2.28 (1.01 - 5.15)**	2.25 (0.93 - 5.43)	**3.50 (1.21 - 10.13)**	**3.05 (1.02 - 9.07)**	2.37 (0.73 - 7.71)	
Hospital	**2.54 (1.26 – 5.11)**	**2.36 (1.16 - 4.80)**	1.66 (0.80 - 3.44)	1.85 (0.82 - 4.17)	**5.35 (2.00 - 14.29)**	**4.37 (1.57 - 12.20)**	2.71 (0.90 - 8.13)	
Other	2.22 (0.71 – 6.87)	2.09 (0.66 - 6.62)	0.60 (0.23 - 1.61)	0.52 (0.18 - 1.55)	1.97 (0.47 - 8.27)	2.00 (0.46 - 8.75)	1.33 (0.31 - 5.73)	
More than 1	**2.56 (0.95 – 6.98)**	2.29 (0.83 - 6.32)	1.88 (0.71 - 4.95)	2.06 (0.76 - 5.60)	1.85 (0.49 - 6.96)	1.59 (0.41 - 6.14)	2.29 (0.54 - 9.64)	
**Visit restrictions**								
No visit restrictions in place	1.00	1.00	1.00		1.00		1.00	
Visit restrictions in place	**2.00 (0.90 – 4.42)**	1.97 (0.76 - 5.12)	1.50 (0.80 - 2.81)		1.70 (0.76 - 3.77)		0.93 (0.46 - 1.89)	
**PPE**								
Enough PPE available	1.00		1.00		1.00		-	
Not enough PPE available	1.21 (0.66 – 1.94)		1.56 (0.60 - 4.05)		1.44 (0.42 - 4.89)			
**Post-death care**								
Allowed to provide post-death care	1.00	1.00	1.00		1.00		1.00	
Not allowed to provide post-death care	**2.28 (1.27 – 4.09)**	**2.00 (1.06 - 3.76)**	1.56 (0.60 - 4.05)		1.51 (0.82 - 2.79)		1.37 (0.65 - 2.92)	

**Regularly exhausted**								
	**T1**		**T2**		**T3**		**T4**	
	Univariate	Multivariate	Univariate	Multivariate	Univariate	Multivariable	Univariate	Multivariate
**Gender**								
Women	1.00		1.00		1.00		1.00	
Men	0.78 (0.36 - 1.67)		0.92 (0.43 - 1.96)		0.63 (0.23 - 1.74)		0.74 (0.26 - 2.09)	
**Age**								
⩽35 years	1.00	1.00	1.00	1.00	1.00	1.00	1.00	
36-45 years	0.98 (0.47 - 2.07)	1.03 (0.47 - 2.29)	**1.95 (0.92 - 4.14)**	2.11 (0.97 - 4.59)	1.22 (0.44 - 3.40)	0.97 (0.29 - 3.17)	2.25 (0.52 - 9.73)	
46-60 years	**0.46 (0.25 - 0.87)**	0.51 (0.26 - 1.04)	1.13 (0.60 - 2.13)	1.41 (0.70 - 2.83)	0.85 (0.37 - 1.94)	1.02 (0.39 - 2.62)	0.69 (0.19 - 2.46)	
>60 years	**0.26 (0.11 - 0.63)**	**0.35 (0.13 - 0.94)**	**0.42 (0.16 - 1.07)**	0.65 (0.24 - 1.76)	**0.35 (0.12 - 1.02)**	0.38 (0.10 - 1.36)	0.34 (0.08 - 1.53)	
**Profession**								
Nurse	1.00		1.00		1.00	1.00	1.00	
Physician	0.72 (0.36 - 1.43)		0.65 (0.31 - 1.35)		1.14 (0.45 - 2.87)	1.18 (0.38 - 3.65)	0.86 (0.33 - 2.25)	
Other	0.83 (0.43 - 1.60)		1.38 (0.78 - 2.44)		**1.91 (0.99 - 3.70)**	**2.20 (1.03 - 4.73)**	0.93 (0.41 - 2.09)	
**Setting**								
Hospice	1.00	1.00	1.00	1.00	1.00	1.00	1.00	1.00
Home	**4.56 (1.70 – 2.07)**	**3.62 (1.27 - 10.28)**	**2.19 (0.87 - 5.49)**	1.71 (0.64 - 4.54)	**4.29 (1.33 - 13.84)**	**4.76 (1.41 - 16.08)**	2.10 (0.61 - 7.20)	2.10 (0.61 - 7.20)
Nursing home	**9.55 (3.74 - 24.44)**	**5.86 (2.19 - 15.68)**	**3.63 (1.49 - 8.83)**	**3.66 (1.41 - 9.48)**	**7.43 (2.34 - 23.61)**	**7.20 (2.16 - 24.04)**	2.55 (0.83 - 7.84)	2.55 (0.83 - 7.84)
Hospital	**8.02 (3.28 - 19.66)**	**6.15 (2.39 - 15.84)**	**3.92 (1.75 - 8.79)**	**3.31 (1.38 - 7.93)**	**9.30 (3.13 - 27.60)**	**8.44 (2.67 - 26.67)**	**3.14 (1.10 - 9.00)**	**3.14 (1.10 - 9.00)**
Other	**4.70 (1.35 - 16.41)**	**4.46 (1.17 - 17.05)**	1.97 (0.74 - 5.22)	1.68 (0.60 - 4.67)	**5.66 (1.34 - 23.88)**	**5.83 (1.18 - 28.91)**	1.57 (0.40 - 6.18)	1.57 (0.40 - 6.18)
More than 1	**4.03 (1.28 - 12.67)**	3.14 (0.97 - 10.23)	**3.01 (1.06 - 8.51)**	2.67 (0.92 - 7.77)	2.03 (0.47 - 8.77)	1.93 (0.43 - 8.67)	1.40 (0.33 - 5.97)	1.40 (0.33 - 5.97)
**Visit restrictions**								
No visit restrictions in place	1.00		1.00		1.00		1.00	
Visit restrictions in place	0.71 (0.32 - 1.56)		1.33 (0.71 - 2.48)		1.49 (0.70 - 3.21)		1.21 (0.61 - 2.41)	
**PPE**								
Enough PPE available	1.00	1.00	1.00		1.00	1.00	-	
Not enough PPE available	**1.68 (1.06 - 2.66)**	**1.95 (1.12 - 3.38)**	1.58 (0.61 - 4.11)		**4.17 (1.07 - 16.28)**	3.43 (0.78 - 15.17)		
**Post-death care**								
Allowed to provide post-death care	1.00	1.00	1.00	1.00	1.00		1.00	
Not allowed to provide post-death care	**1.91 (1.04 - 3.53)**	1.78 (0.87 - 3.64)	**1.78 (1.09 - 2.93)**	1.89 (1.09 - 3.27)	0.92 (0.51 - 1.67)		1.40 (0.67 – 2.93)	

*N= up to four observations from 276 respondents; based on Generalized Estimated Equation

Bold values in the univariate analysis indicated OR’s that significantly differ from 1.00 (*p*<0.10)

Bold values in the multivariate analysis indicated OR’s that significantly differ from 1.00 (*p*<0.05)

Furthermore, multivariable analyses per time period shows that there are some differences in whether characteristics of HCPs and COVID-related care were associated with signs of distress per time period. For feeling more stressed than usual setting was associated in both T1 and T3. In both time periods compared to hospice, HCPs in other settings were more likely to feel more stressed. In T1 this was most likely in home settings (OR 3.63) and in T3 this was most likely in hospital (OR 4.20). Only in T1 not being able to provide post-death care was associated with being more stressed than usual (OR 2.00)

For all four time periods, it was more likely for HCPs to feel exhausted in all settings compared to in hospice facilities. The odds ratios were highest in T3, ranging from 4.76 for the home setting to 8.44 for the hospital. Of the COVID-related care restriction no PPE being available was associated with regularly feeling exhausted in T1 (OR 1.95), and not being allowed to provide post-death care was associated with regularly feeling exhausted in T2 (OR 1.89).

## Discussion

This longitudinal study measures signs of distress of healthcare providers (HCPs) who provided end-of-life care during the initial 18 months of the COVID-19 pandemic. Although reported distress was highest in the first period, during the first wave of COVID-19 pandemic, HCPs reported signs of distress in all four time periods. Being more stressed than usual and being regularly exhausted were the most common signs of distress. HCPs working in nursing homes and hospitals were more likely to experience signs of distress, compared to HCPs working in hospice facilities, during the whole period of 1.5 years. When HCPs were restricted in providing post-death care, they were more likely to feel more stressed than usual and find their work more often emotionally demanding.

### Distress overtime

Throughout the first 1.5 year of the pandemic gradually less HCPs experienced signs of distress, to a time point where there was a significant decline from the beginning of the pandemic, except for exhaustion. At all time points during the 18 months of our study, approximately four out of ten HCPs experienced exhaustion. This percentage applies to HCPs of all settings, but our GEE analysis shows differences between settings. Compared to HCPs in hospice facilities, HCPs in hospitals (OR 3.90) and in nursing homes (OR 3.92) were more frequently regularly exhausted. Therefore, the percentages of HCPs experiencing exhaustion is even higher in these specific settings compared to the overall average. A Canadian study among nursing staff in hospitals during the pandemic confirms the high exhaustion rate over the course of the pandemic in hospitals, reaching almost 60% in the spring of 2021 [18].

Despite a decrease in the percentage of most signs of distress over time, with the exception of exhaustion, still 19% (up unto 42%) of HCPs experienced at least one sign of distress 1.5 years after the onset of the pandemic. To understand the considerable prevalence of experiences of distress and increased support needs of HCPs related to end-of-life care during and after the pandemic, comparison to experienced distress before the pandemic is important. In two specific questions included in our research, we already asked respondents to compare their well-being to the period before the onset of the pandemic, so we do know respondents were more stressed and needed more emotional support. Unfortunately, studies on distress among Dutch HCPs in palliative care are scarce and comparison on distress is difficult. Regarding exhaustion, a study performed just before the pandemic shows that around 8% of providers had high to very high exhaustion levels, while 55% scored at a medium level [2]. However, it is difficult to draw conclusions about differences between exhaustion levels before and during the pandemic based on this study and the current study, because of the variation in outcome measures.

Our results show that even when most restrictions were lifted and the number of COVID-19 cases declined, many HCPs in our study still experienced distress to some extent. Since prolonged emotional and interpersonal stress on the job can lead to burnout [19], even after the pandemic HCPs may be susceptible to developing a burnout because of what they endured during the pandemic. This might also challenge the health care systems given the current and projected shortages of staff. A review on experiences of women in healthcare highlights the importance of recognizing that women's well-being was particularly affected during the pandemic. Therefore, a gender-specific approach is important in addressing the mental health issues of healthcare providers during a health care crisis [20].

### Distress in different settings

For all signs of distress, HCPs in hospitals and nursing homes were more likely to experience distress compared to nurses in hospices. This could be attributed to notable differences in COVID-19 infection prevention measures and their application in various settings. We know from another study that HCPs working in hospitals and nursing homes were less flexible to deviate from those restrictions and make individual decisions for their patients and shape their caregiving approach compared to HCPs in home care and hospices [1].

### The influence of the preventative measures on distress

Healthcare delivery deviated from the norm during the first 18 months COVID-19 pandemic, since HCPs were confronted with restrictions. This study illustrates that especially in the initial two periods, the shortage of PPE and the inability to provide post-death care impacted the well-being of HCPs.

A Brazilian study also identified an association between emotional distress and insufficient access to PPE during the initial months of the pandemic [21]. Various reasons could account for this. First, a shortage of PPE can lead healthcare providers to be more concerned about their own health, as well as the well-being of their loved ones and patients [12, 22, 23]. Furthermore, other studies indicate that insufficient PPE can compel HCPs to alter their caregiving approach, necessitating choices in patient contact moments [1, 2, 24]. Each interaction consumes PPE, prompting decisions such as refraining from in-person visits to conserve resources.

Regarding post-death care, HCPs faced limitations in delivering care according to standard practices, as protocols prohibited actions like taking care of the bodies of the deceased patients, as is also seen in other studies [1, 12, 25]. Part of post-death care involves the ritual surrounding the passing. In a study on post-death care, nurses express the importance of honoring and showing respect for the deceased in this manner [26]. Furthermore, providing post-death care also allows HCPs to say goodbye to their patients [26] and restrictions in providing post-death might hinder this process.

Both factors share a common element that manifested in various ways during the pandemic: the inability to provide care as healthcare providers desired. Both the shortage of PPE and the restrictions in providing post-death care can lead to healthcare providers delivering different care than they want to. This misalignment may not always align with their professional moral values and therefor may cause moral distress. In our study, we did not directly inquire about moral distress. However, existing literature indicates a connection between COVID-19-related care restrictions and their impact on the emotional well-being of healthcare providers [11, 27].

Factors that did not contribute to distress in our study include visiting restrictions. One plausible explanation could be the exploration of alternative means to facilitate contact between patients and their loved ones, such as video calling, thereby minimizing the impact on the well-being of healthcare providers. Additionally, other studies suggest that, in certain situations, visit restrictions provided some HCPs with a sense of relief, as they had more time for patients themselves, since they spent less time on attending to the needs of the patient’s relatives.

Moreover, the analysis for each time period in our study indicates that the inability to provide post-death care and the shortage of PPE had an impact on the distress of healthcare providers only in T1 and T2, even though restrictions were still often in place in T3 and T4. This might suggest that healthcare providers may have adapted better to the restrictions in a somewhat later phase of the pandemic.

### Strengths and limitations

It is possible that respondents that were in severe emotional distress did not complete the (follow-up) questionnaires, resulting in a sample that predominantly represents the respondents that were doing (relatively) well. This could lead to an underestimation of distress in HCPs. Moreover, there might have been recall bias when the first questionnaire was conducted because it contained questions about an earlier time period. However, the start of the pandemic and thereby the first period was an exceptional period, so people might remember very well how they felt during that time. Furthermore, the question if visit restrictions were in place was asked in a general way; we cannot say anything about the severity of the visit restrictions and for this reason, results may be over- or underestimated. A strength of our study is that all care settings and different professions with care for both COVID-19 and non-COVID-19 patients were included. In this way our study offers a broad perspective. Furthermore, the longitudinal aspect of our study is strength, as we have data up to 18 months after the beginning of the pandemic.

## Conclusion

In conclusion, we found that a large amount of HCPs reported signs of distress during all periods of the initial 18 months COVID-19 pandemic. Most signs of distress were reported in the first period, but still a substantial part of HCPs showed signs of distress after 1,5 years. This means that the well-being of healthcare providers is at stake. A cause of distress appears to be that healthcare providers cannot provide the care they desire. In this case, it was partly due to the pandemic, but presently, this remains an important and relevant finding, as high workload can sometimes force healthcare providers to make choices about how they provide care. Given that this can cause prolonged stress and this can lead to burnout (and HCPs leaving their current positions), it is now especially important to continue observing the long term developments of the well-being of our healthcare providers in palliative care and provide timely and adequate support where needed.

### Supplementary Information

**Supplementary Material 1.**

## Data Availability

The datasets used and/or analysed during the current study are available from the corresponding author on reasonable request.
