# Supplementary material for: Distress among healthcare providers who provided end-of-life care during the COVID-19 pandemic: a longitudinal survey study (the CO-LIVE study)
Source: BMC Palliat Care. 2024 May 28;23:134. doi: 10.1186/s12904-024-01446-y (PMC11131237; doi:10.1186/s12904-024-01446-y)
Supplement: Supplementary file 1 — Supplementary Material 1. [file 12904_2024_1446_MOESM1_ESM.docx]

| **Appendix 1: Original (translated) and recoded questions in the questionnaire** | | |
| --- | --- | --- |
| **Variable** | **Original question** | **Recoded** |
| **Gender** | **What is your gender?**   - Male - Female - Other - I would rather not say |  |
| **Age** | **What is your age?** | **Categorized**  *⩽35 years*  *36-45 years*  *46-60 years*  *>60 years* |
| **Profession** | **In what role were you caring for patients?**  Nursing assistant  Nurse  Physician assistant  General practitioner  Elderly care physician  Other medical specialty, namely:  ___________________________________  Other care professional, namely:  __________________________________  Volunteer | **Categorized**   - Nurse   *Nursing assistant, nurse, physician assistant*   - Physician  *General practitioner, elderly care physician, physician with a different specialism* - Other  *Other healthcare professional, volunteer* |
| **Setting** | **In which setting did you provide care?**  *(more than one answer possible)*  At home / community  In a hospital  At an ICU  At a ward for Corona patients (no ICU)  At another ward  In a care home / nursing home  At a ward for Corona patients  At another ward  In a hospice facility (not specifically for Corona patients)  Other (please specify): _______________________________ | **Categorized**   - Home   *At home/community*   - Hospital   *In a hospital (ICU/at ward for COVID patients, at another ward)*   - In a care home / nursing home   *(At a ward for COVID patients, at another ward)*   - Hospice facility   *For COVID patients, not for COVID patients*   - Other   *Other*   - More than one |
| **Visit restrictions** | **Where there measures in place regarding the visitation of loved ones at the place where you worked when it became clear that the patient was nearing death?**  *(more than one answer possible)*  No  Yes, a maximum number of persons per day, namely:___________  Yes, a maximum amount of time, namely maximum _____ hours a day.  Yes, it changed/differed during the period, namely: _____________  Yes, other namely _____________________ | **Dichotomized**   - Restrictions   *A maximum number of persons per day, a maximum amount of time, it changed/differed during the period*   - No restrictions   *No* |
| **Enough PPE** | **Was there enough personal protective equipment during the first wave?**  Yes  No, not always  No, not for everyone who needed it  No | **Dichotomized**   - Enough PPE   *Yes*   - Not enough PPE   *Not always, not for everyone who needed it, no* |
| **Allowed to provide post-death care** | **What measures/restrictions where there after the death of a patient during the first wave?**  (more than one answer possible)  None  The deceased patient was taken away immediately  Me and my colleagues were not allowed to care for the deceased  Me and my colleagues could not say goodbye in the usual way  The family could not say goodbye  Other, namely (please specify) | **Dichotomized**   - Were allowed to provide post-death care - Were not allowed to provide post-death care |
| **Statements about distress** | Please rate to what extent you agree with the follow statements when you think about how you felt during the last period**?**  - I felt more stressed than usual - My work was emotionally demanding for me - My work was physically demanding for me - I regularly felt exhausted.  Strongly disagree  Disagree  Neutral  Agree   Strongly agree  Don’t know | **Dichotomized**   - Disagree   *Strongly disagree, disagree*   - Neutral *Neutral* - Agree   *Agree, strongly agree* |
| **Statement about support** | How much emotional support did you need first wave?  ☐ More than usual  As much as usual  ☐ Less than usual |  |
